# IDO1 correlates with the immune landscape of head and neck squamous cell carcinoma: a study based on bioinformatics analyses

**DOI:** 10.3389/froh.2024.1335648

**Published:** 2024-04-26

**Authors:** Georgia Vasiliki Gkountana, Lezhou Wang, Martina Giacomini, Aini Hyytiäinen, Krista Juurikka, Tuula Salo, Ahmed Al-Samadi

**Affiliations:** ^1^ Department of Oral and Maxillofacial Diseases, Clinicum, Faculty of Medicine, University of Helsinki, Helsinki, Finland; ^2^ Translational Immunology Research Program (TRIMM), Faculty of Medicine, University of Helsinki, Helsinki, Finland; ^3^ Research Unit of Population Health, Faculty of Medicine, University of Oulu, Oulu, Finland; ^4^ Medical Research Center Oulu, Oulu University Hospital, University of Oulu, Oulu, Finland; ^5^ Faculty of Biochemistry and Molecular Medicine, University of Oulu, Oulu, Finland; ^6^ Institute of Dentistry, School of Medicine, Faculty of Health Sciences, University of Eastern Finland, Kuopio, Finland

**Keywords:** head and neck squamous cell carcinoma, IDO1, immune cells, prognostic marker, immune checkpoint

## Abstract

**Background:**

Head and neck squamous cell carcinoma (HNSCCs) is a common cancer type with a high mortality rate and poor prognosis. Recent studies have focused on the role of immune checkpoints in HNSCC progression and in their potential use as prognostic markers and immunotherapeutic candidates. Some immune checkpoints, such as PD-1 and PD-L1, have been studied thoroughly in HNSCC. Other molecules, such as indoleamine 2,3-dioxygenase 1 (IDO1), have been investigated minimally.

**Methods:**

IDO1 expression, prognostic potential, and association with the immune profile of HNSCC were explored using online databases, including GEPIA, UALCAN, TIMER2.0, cBioPortal, and LinkedOmics, which utilize TCGA datasets and are freely available for use. For validation purposes, seven pairs of primary and metastatic HNSCC were immunostained for IDO1.

**Results:**

Our analysis revealed significantly higher expression of IDO1 in HNSCC, especially in HPV+ SCCs compared with healthy control tissue. However, IDO1 expression showed weak to no prognostic potential for overall and disease-free survival in HNSCC. IDO1 expression in HNSCC was positively correlated with several immune-related molecules, including most of the immune checkpoints. Additionally, GO enrichment analysis revealed that several immune-related pathways are positively correlated with IDO1 expression in HNSCC, such as response to type I interferon and lymphocyte-mediated immunity pathways. Finally, IDO1 expression positively correlated with infiltration of most of the immune cells in HNSCC, such as CD4+ T cells, CD8+ T cells, M1 and M2 macrophages, dendritic cells, and B cells.

**Conclusion:**

IDO1 expression is closely correlated with the immune profile of the HNSCC. This observation should be explored further to elucidate the potential of targeting IDO1 as a novel immunotherapeutic approach for HNSCC.

## 1 Introduction

Head and neck squamous cell carcinoma (HNSCC) is the eighth most common cancer type worldwide (1). HNSCC affects the mucosal surfaces of the oral cavity, nasal cavity, pharynx, and larynx and is associated with several risk factors, including alcohol consumption, tobacco use, and some viral infections, such as human papillomavirus (HPV) and Epstein-Barr virus (2, 3). HNSCC is currently treated with surgery with or without radiotherapy, chemotherapy, or targeted therapy (4). Additionally, nivolumab and pembrolizumab (anti-PD-1 antibodies) have recently been approved for treatment of recurrent or metastatic HNSCC (5).

As in all cancer types, HNSCC uses a variety of mechanisms to evade the immune system and to promote tumor growth and metastasis (6). One of these mechanisms is promoting immunosuppression, for example by stimulating the differentiation of suppressive immune cells (7). In fact, HNSCC patients have increased levels of T regulatory cells (Tregs) within the tumor-infiltrating lymphocyte (TIL) fraction, and immune responses of effector T cells are often defective (7, 8). Moreover, overexpression of immune checkpoints on the surface of T cells is another mechanism of immune evasion in HNSCC (9). The main immune checkpoint pathways in HNSCC include PD-1/PD-L1, cytotoxic T lymphocyte antigen 4 (CTLA-4), T-cell immunoglobulin mucin 3 (TIM-3), lymphocyte activation gene 3 (LAG-3), T-cell immunoglobulin and immunoreceptor tyrosine-based inhibitory motif (TIGIT), and indoleamine 2,3-dioxygenase 1 (IDO1) (9, 10).

IDO1 is a catabolic enzyme that converts tryptophan (Trp) into kynurenine in peripheral tissues (11) and is an immune-checkpoint molecule that is highly expressed in many cancers. IDO1 overexpression is usually associated with poor prognosis (12). In the tumor microenvironment (TME), IDO1 is expressed by cancer and stromal cells (such as tumor-associated macrophages and fibroblasts), endothelial cells, and some immune cells [such as dendritic cells and other antigen presenting cells (APC)] (11–13). IDO1 overexpression leads to Trp depletion, which in turn leads to inhibition of the mammalian target of rapamycin complex (mTORC) pathway and activation of the stress-response general control over nonderepressible (GCN2) kinase in tumor-infiltrating T cells, which both lead to immunosuppression (11). In addition, aryl hydrocarbon receptor (AhR) on several immune cell types is also activated, further promoting a suppressive TME (11). Furthermore, IDO1 is positively correlated to levels of TILs in HNSCC. Some reports identified IDO1 as a potential prognostic marker for HNSCC (14).

The role of IDO1 in HNSCC metabolism and immunosuppression is not fully understood. More data are needed to understand the clinical relevance of IDO1 in immunotherapeutic approaches for HNSCC and its potential as a biomarker. Moreover, knowledge on IDO1 in HNSCC may reveal new pathways that could be targeted therapeutically or used for prognostication. Further elucidating the crosstalk between the immune system, cancer cells, and TME-related molecules may lead to a better understanding of disease development and progression. Several databases, such as TIMER, UALCAN, and cBioPortal contain an abundance of multi-omics patient data for different types of cancers. Here, we sought to analyze data acquired from different databases to investigate IDO1 expression in HNSCC tumors and its correlation with patient survival, drug response, and tumor immune profile. Our analysis revealed that IDO1 is correlated with the immune landscape of HNSCC patients through a positive correlation with immune-related genes and immune-cell infiltration.

## 2 Methods

### 2.1 IDO1 gene expression analysis in normal tissues and HNSCC samples and its correlation with patient survival

Gene expression analysis of IDO1 in 520 HNSCC tumor samples from The Cancer Genomic Atlas (TCGA) and 44 adjacent normal tissue samples was performed using the TIMER2.0 database (15). TIMER2.0 provides gene-expression level data represented in a log2 transcripts per million (TPM) scale. The UALCAN platform (http://ualcan.path.uab.edu) was used to assess the correlation between IDO1 gene expression, illustrated as TPM, and patients' clinical and pathological characteristics, such as tumor stage and grade and patient age and gender (16, 17).

Single-cell RNA-seq data for eight distinct cell types (fibroblasts, macrophages, B, T, dendritic, cancer, endothelial, and mast cells) in HNSCC were retrieved from Gene Expression Omnibus database with the accession code GSE103322 (https://www.ncbi.nlm.nih.gov/geo/query/acc.cgi?acc=GSE103322). IDO1 expression levels in different cell populations in both primary tumor and lymph node metastasis (LNM) were analyzed and visualized using R (version 4.2.2) with dplyr, reshape2, data.table, tidyverse, ggplot2, ggpubr, hrbrthemes, viridis, and scales packages. Statistical significance in IDO1 expression in different cell types between primary tumor and LNM was assessed with Student's *t*-test. *p*-value < 0.05 was considered statistically significant.

Overall survival (OS) and disease-free survival (DFS) of HNSCC patients were evaluated in correlation to tumoral IDO1 expression using GEPIA (http://gepia.cancer-pku.cn) webtools and data obtained from TCGA Program project consisting of 518 HNSCC samples. The median of IDO1 expression levels was used as a group cutoff to determine high- and low-IDO1 expressing groups.

### 2.2 Immunostaining

Seven pairs of primary and metastatic HNSCC samples were collected at the Oulu University Hospital. The data inquiry was approved by the Finnish Medicines Agency (FIMEA). The use of patient material for this study was approved by the Northern Ostrobothnia Hospital District Ethics Committee (statement 101/2020). Detailed patient data are presented in Table 1.

**Table 1:** Baseline characteristics of patients with HNSCC.

Patient clinical data	No. of patients (%)
	*n*tot = 7
Age, years	
<60	4 (57.1)
≥60	3 (42.9)
Range	31–72
Mean	54.9
Median	54
Sex	
Male	6 (85.7)
Female	1 (14.3)
Tumor grade	
Moderate (II)	4 (57.1)
Poor (III)	3 (42.9)
Tumor stage	
T1–T2	0 (0)
T3–T4	7 (100)
Lymph node metastasis	
N1	7 (100)
N0	0 (0)
Treatment	
Surgery and radiotherapy	3 (42.9)
Surgery, radio- and chemotherapy	3 (42.9)
Missing	1 (14.2)

Samples were cut into 4 µm thick sections and deparaffinized. Antigen retrieval was carried out in citrate buffer using a microwave oven (MicroMED T⁄T Mega Histoprocessing Labstation; Milestone Srl, Sorisole, Italy). Afterwards, the slides were subjected to immunohistochemical (IHC) staining. The slides were incubated in Dako Peroxidase blocking agent (Agilent DAKO, Santa Clara, USA) for 15 min. After washes in PBS 3 × 5 min, the slides were incubated in 10% normal goat serum (Vector Laboratories, Burlingame, CA, USA) for 1h at room temperature (RT). Normal serum was blotted away, and the slides were incubated with 1:50 monoclonal Alexa Fluor-488 rabbit anti-human IDO1 primary antibody (Abcam, Cambridge, UK) overnight at +4°C. After washes, the slides were incubated with 1:300 biotinylated goat anti-rabbit secondary antibody (Vector Laboratories) in 2% BSA-PBS and 1% normal goat serum for 1 h at RT. After washes, the slides were incubated with avidin–biotin–peroxidase complex (Vectastain Elite ABC kit; Vector Laboratories) for 1 h at RT. After washes, the slides were incubated in 3,3′-diaminobenzidine tetrahydrochloride (DAB) chromogen (Agilent DAKO) for 10 min at RT. After washes in PBS 1 × 5 min and dH2O 2 × 5 min, the slides were incubated with Mayer's hematoxylin (Sigma-Aldrich, St. Louis, MI, USA) for 2 min at RT. After washing under running water for 10 min, the slides were dehydrated and cleared in xylene, mounted with J.T.Baker UltraKitt (Avantor, Radnor, PA, USA) and imaged.

Another batch of deparaffinized and antigen retrieved slides was used to perform double immunofluorescence (IF) staining. The slides were incubated with Intercept PBS Blocking Buffer (LI-COR, Lincoln, NE, USA) for 30 min at RT. The blocking buffer was blotted away, and the slides were incubated in 1:200 monoclonal mouse anti-human CD163 primary antibody (Leica, Deer Park, IL, USA) and 1:10,000 Hoechst (Thermo Scientific, Waltham, MA, USA) overnight at +4°C. After washes in PBS 3 × 5 min, the slides were incubated in 1:200 polyclonal Alexa Fluor-568 donkey anti-mouse secondary antibody (Invitrogen, Waltham, MA, USA) for 1h at RT. After washes in PBS 3 × 5 min, the slides were incubated in 1:50 monoclonal Alexa Fluor-488 rabbit anti-human IDO1 primary antibody (Abcam, Cambridge, UK) in PBS overnight at +4°C. After washes in PBS 3 × 5 min the slides were mounted using SlowFade diamond antifade (Invitrogen) and imaged.

Stained tissues were analysed and photographed using Leica DM6000 B/M light microscope connected to a digital camera (DFC420 and DFC365FX; Leica Microsystems, Wetzlar, Germany).

### 2.3 Analysis of IDO1 genetic alteration and DNA methylation

We used cBioPortal (https://www.cbioportal.org/) to investigate IDO1 genetic alterations and their possible effect on HNSCC patient survival (18, 19). For this purpose, the TCGA Firehose Legacy dataset was used and 504 HNSCC tumor samples were screened for mutations and copy number alterations (CNAs). Correlation between IDO1 alteration and patient survival was also explored.

The UALCAN database was employed to evaluate IDO1 promoter methylation levels in HNSCC and its association with patient clinical and pathological characteristics, such as tumor stage and grade and patient age and gender, in 528 TCGA HNSCC tumor samples and 50 normal tissue samples.

### 2.4 Correlation analysis of IDO1 expression and drug sensitivity on HNSCC cell lines

The association between IDO1 expression and drug sensitivity in HNSCC cell lines was assessed using the “Genomics of Drug Sensitivity in Cancer (GDSC) gene expression—drug sensitivity correlations” online tool provided by Tableau Public (https://public.tableau.com/profile/jason.roszik#!/vizhome/CCLE_GDSC_correlations/CCLE_GDSC). This dataset contains gene-expression and drug-screening data for 40 HNSCC cancer cell lines originating from the GDSC project (20).

### 2.5 IDO1 co-expression genes and gene set enrichment analysis

The LinkedOmics (http://www.linkedomics.org/) database was used to identify genes that are co-expressed with IDO1 in 520 TCGA HNSCC tumor samples, based on TCGA RNA sequencing data (21). IDO1 co-expressed genes are presented in a volcano plot produced using Spearman's correlation test. Moreover, Gene Ontology (GO) analysis for biological process, cellular component, molecular function, and KEGG pathway analysis were performed using the gene set enrichment analysis (GSEA) tool of LinkInterpreter. For the GSEA, the following parameters were chosen: false discovery rate (FDR) < 0.05 as rank criteria; minimum number of genes 3; and 1,000 simulations.

Additionally, the “Cancer Exploration” tool of TIMER2.0 was utilized to define the association between IDO1 and several immune checkpoints expression, including ADORA2A, B7-H1 (PD-L1), CD276, CTLA4, C10ORF54, KIR2DL1, KIR2DL3, KIR2DS4, KIR3DL1, KIR3DL2, KIR3DL3, KIR3DP1, KIR3DX1, LAG3, PD-1, and VTCN1 in 520 TCGA HNSCC tumor samples.

### 2.6 Correlation analysis of IDO1 expression and immune cell infiltration

Lastly, the “Immune Association” tool of TIMER2.0 was used to assess the correlation between IDO1 expression and immune infiltrates in 520 TCGA HNSCC tumor samples (15). The xCELL (https://xcell.ucsf.edu/) estimation tool was used as it contains gene-expression data for a large number of immune cells (22). Tumor purity adjustment was applied for the analysis.

## 3 Results

### 3.1 IDO1 expression is higher in HNSCC tumors compared to normal tissue and high expression predicts better overall survival for HNSCC patients

**Figure 1:**
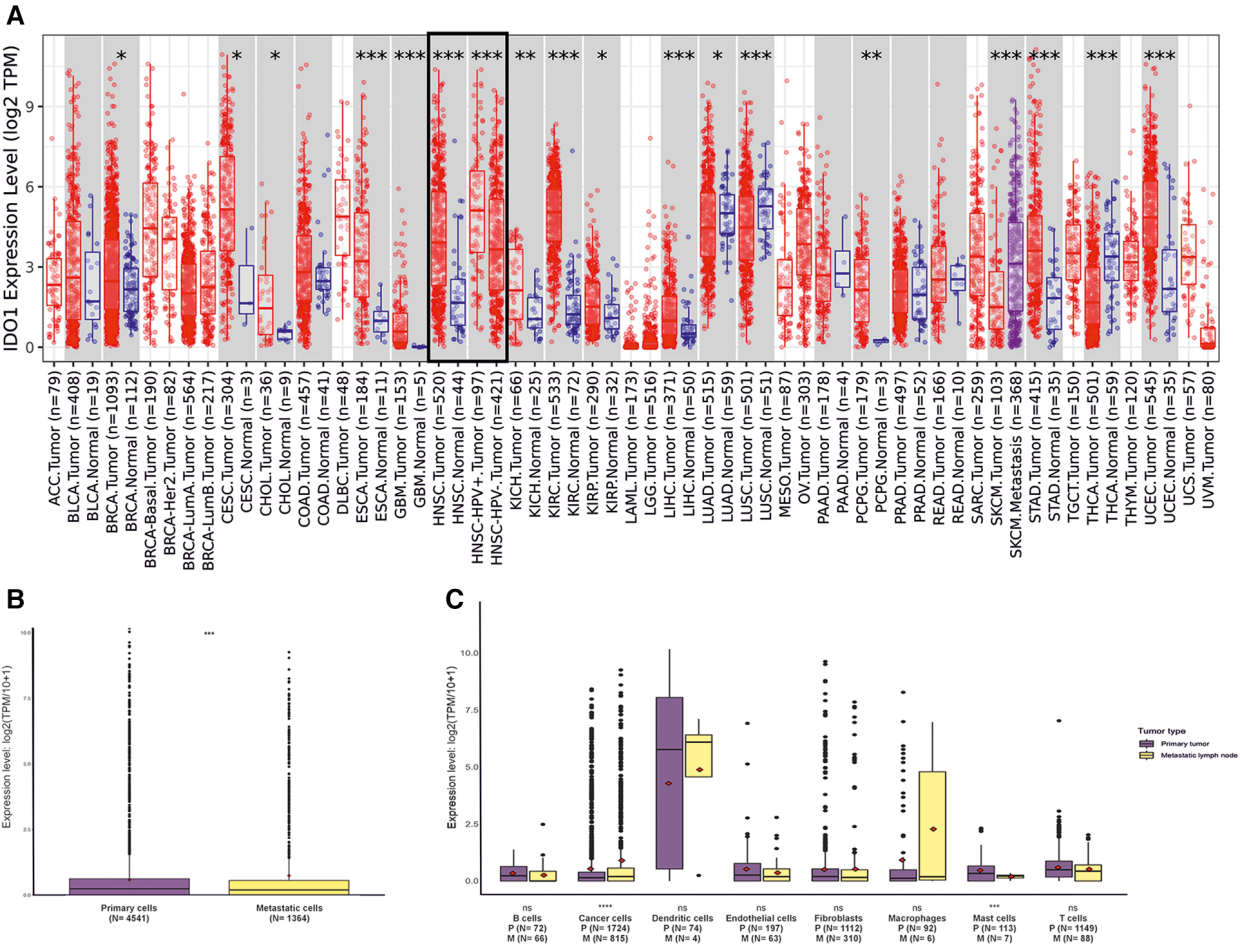
IDO1 expression levels in different tumors, matched normal tissues, and in various cell populations in HNSCC primary tumors and metastatic lymph nodes. (**A**) IDO1 expression in different tumors and their matched normal tissues. Statistical significance computed by the Wilcoxon test is provided by TIMER2.0. (**B**) IDO1 expression in primary and metastatic lymph node tumors. (**C**) Comparison of IDO1 expression levels in different cell types between primary tumors and metastatic lymph node sites. Statistical significance was assessed with Student's *t*-test. *indicates *p*-value < 0.05; **p-value < 0.01; ***p-value < 0.001; ****p-value < 0.0001. HNSCC, head and neck squamous cell carcinoma; HPV, human papilloma virus; TPM, transcripts per million; P, primary tumor; M, metastatic lymph node.

Gene expression analysis using TIMER2.0 revealed significantly higher (*p* < 0.001) IDO1 expression in HNSCC tumor samples (*n* = 520) than in the adjacent normal tissues (*n* = 44, Figure 1A). In addition, when HNSCC samples were stratified according to HPV− (*n* = 421) and HPV+ (*n* = 97) status, a significantly higher (*p* < 0.001) expression of IDO1 was observed in HPV+ compared to HPV− tumors (Figure 1A).

To investigate the cellular sources of IDO1 expression in HNSCC tumors, we analyzed a single-cell RNA-seq dataset taken from the study of Puram et al. (23). IDO1 expression in the metastatic lymph node tumors was slightly but significantly (*p* < 0.001) higher than in the primary tumors (Figure 1B). Interestingly, cancer cells in metastatic lymph nodes express significantly (*p* < 0.0001) higher levels of IDO1 than cancer cells in the primary tumor (Figure 1C). On the other hand, mast cells in metastatic lymph nodes express lower levels (*p* < 0.001) of IDO1 than the mast cells in the primary tumor. However, there were only seven mast cells analyzed in the metastatic lymph nodes compared with 113 cells in the primary tumor (Figure 1C). No other statistically significant difference was observed for the remaining cell types (i.e., B cells, dendritic cells, endothelial cells, fibroblasts, macrophages, and T cells), (Figure 1C). Macrophages and dendritic cells showed the highest level of IDO1 expression compared with other cells (Figure 1C).

**Figure 2:**
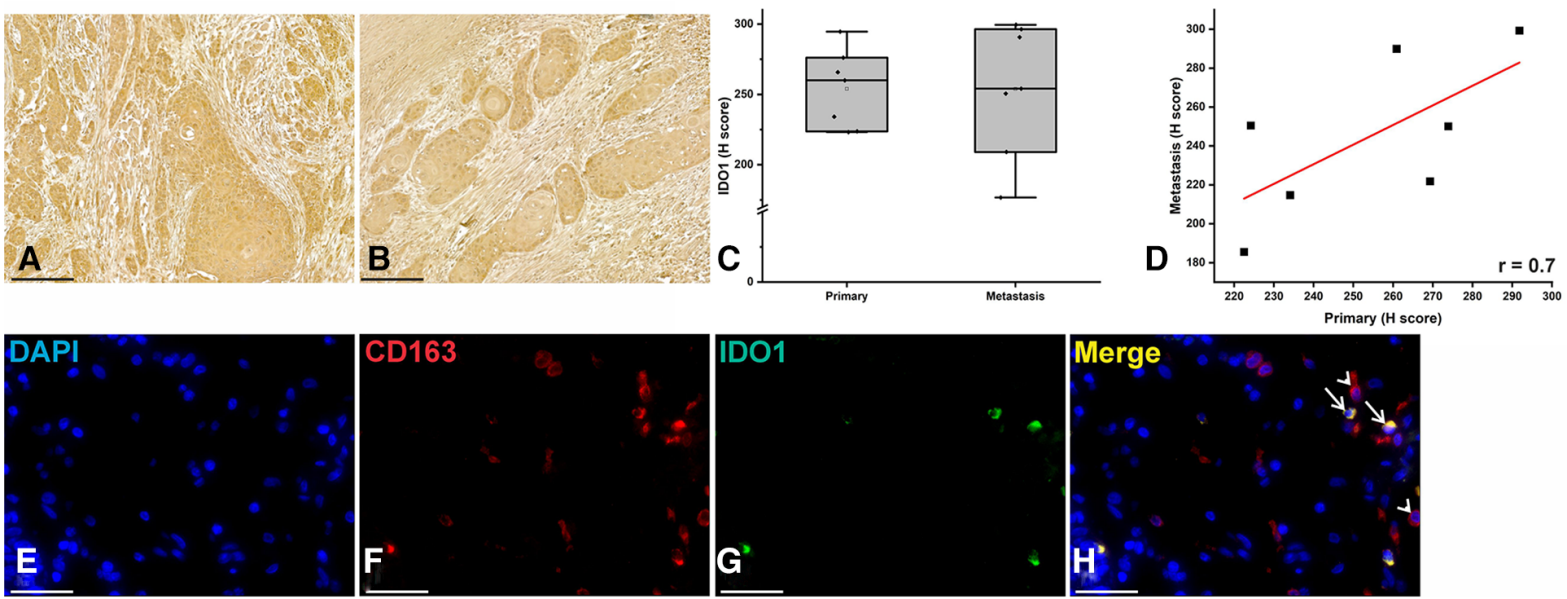
IDO1 immunostaining in HNSCC samples. (**A–C**) Immunohistochemical staining revealed similar staining intensity of IDO1 between primary and metastatic HNSCC cells. (**D**) Pearson correlation showed a moderate correlation of IDO1 expression between the primary and metastatic HNSCC cells. (**E–H**) Double immunofluorescence staining showed double positive cells for IDO1 (green) and macrophage marker CD163 (red).

In order to validate the single-cell RNA-seq dataset, we did IHC staining of IDO1 on seven pairs of primary and metastatic HNSCC samples (Figures 2A,B). Primary and metastatic cancer cells showed similar levels of IDO1 expression (Figure 2C). Interestingly, we found a moderate correlation (*r* = 0.07) of IDO1 expression between the primary and metastatic cancer cells (Figure 2D). Additionally, we conducted double IF staining of IDO1 and macrophage marker (CD163, Figures 2E–H). Similar to the single-cell RNA-seq dataset, several macrophages were positive for IDO1 indicating that macrophages are one source of IDO1 in HNSCC (Figures 2E–H).

**Figure 3:**
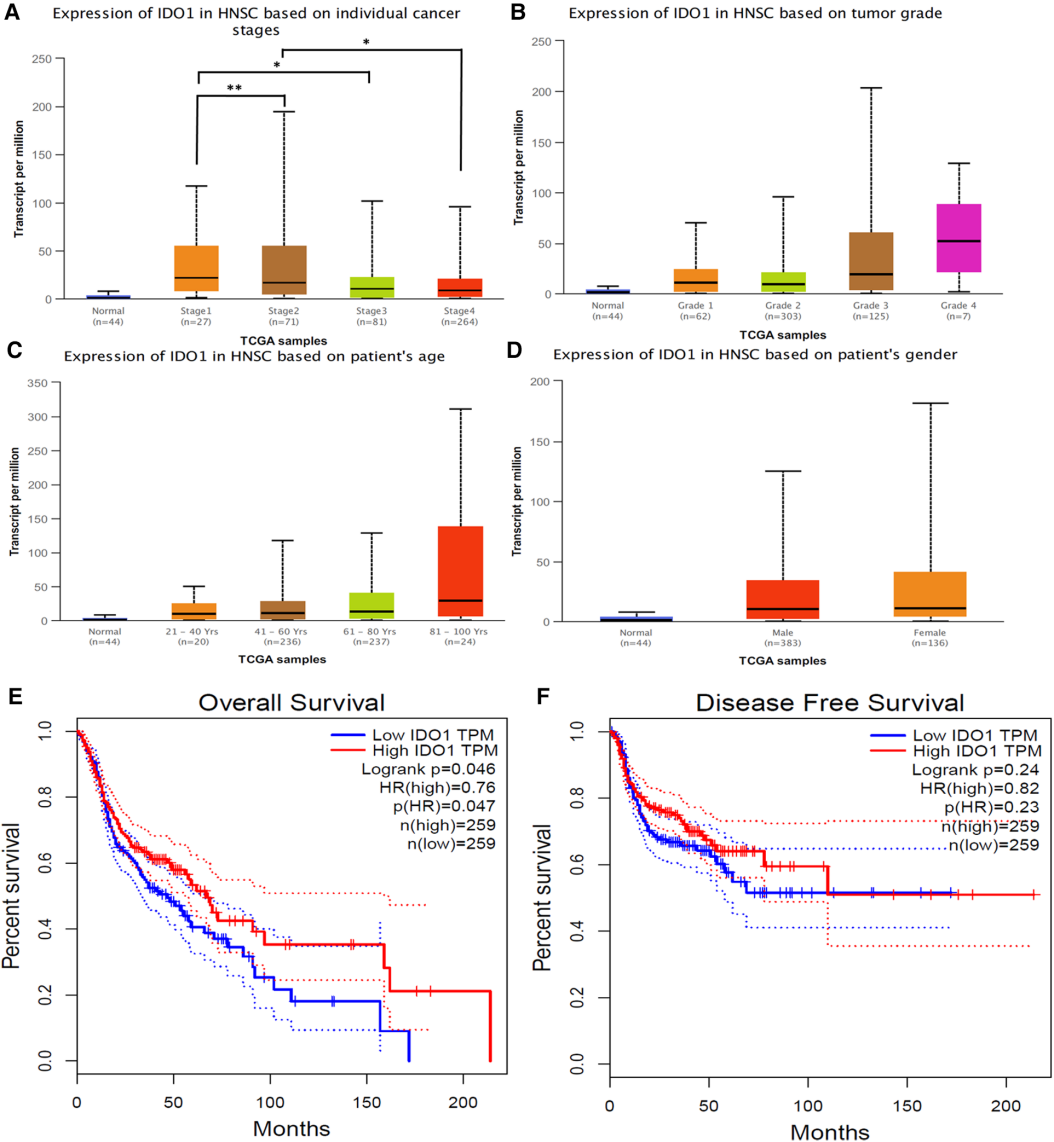
Correlation between IDO1 expression and clinicopathological characteristics of HNSCC patients. (**A**) tumor stage, (**B**) tumor grade, (**C**) patient age, (**D**) patient gender. Statistical significance is provided by UALCAN and is represented by the number of stars; *p-value < 0.05; **p-value < 0.01. Normal samples were not included in the statistical analysis. (**E**) Analysis of IDO1 expression in correlation to HNSCC patient overall survival and (**F**) disease-free survival. HNSCC, head and neck squamous cell carcinoma; HR, hazard ratio; TPM, transcripts per million.

Investigating IDO1 expression against clinicopathological characteristics of HNSCC patients using the UACLAN database revealed a significant decrease in IDO1 expression with increasing tumor stage (Figure 3A). On the other hand, IDO1 did not have a significant correlation with the other clinical and pathological characteristics of HNSCC patients, such as tumor grade (Figure 3B), patient age (Figure 3C), and patient gender (Figure 3D). However, we observed a trend (*p* = ns) for increased IDO1 expression with increasing tumor grade (Figure 3B).

We examined the association between IDO1 expression and OS and DFS of HNSCC patients using GEPIA. Univariate analysis revealed a significant correlation between IDO1 expression and patient OS (*p* = 0.047, Figure 3E); HNSCC patients with higher IDO1 expression had a better OS than patients with lower IDO1 expression. However, no significant correlation was observed between IDO1 expression and patient DFS (Figure 3F). Multivariate analysis was not performed on this data.

### 3.2 IDO1 genetic alteration and DNA methylation in HNSCC

**Figure 4:**
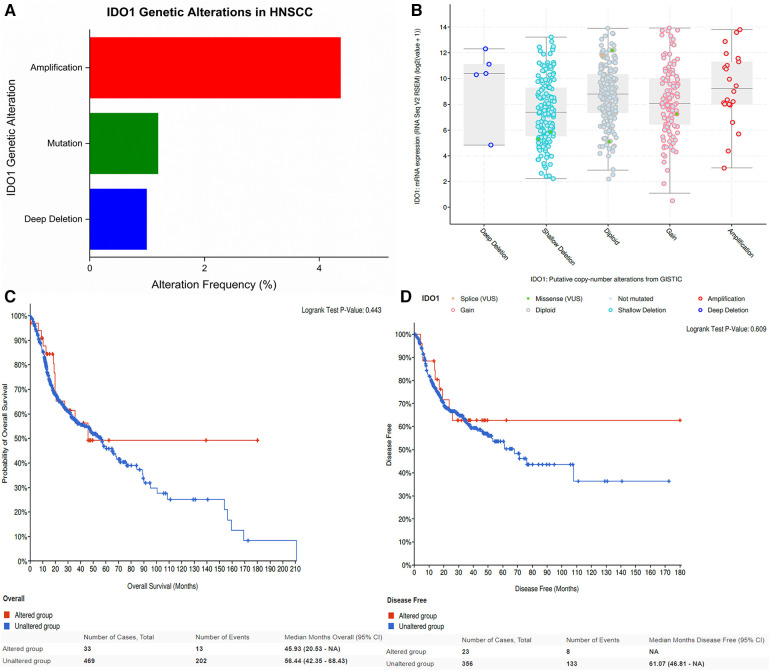
IDO1 genetic alterations in HNSCC and their effect on patient survival. (**A**) Frequency and types of IDO1 genetic alterations in HNSCC tumors. (**B**) Correlation between IDO1 mRNA expression and copy-number alteration in HNSCC tumors. (**C**) Overall survival comparison between IDO1-altered and IDO1-unaltered patient groups. (**D**) Disease-free survival comparison between IDO1-altered and IDO1-unaltered patient groups. HNSCC, head and neck squamous cell carcinoma.

Next, we examined genetic alterations in the IDO1 gene in HNSCC tumors using cBioPortal, which revealed that IDO1 was altered in 33 out of 504 HNSCC tumor samples (6.5% of the samples, Figure 4A). The most common alteration was amplification in 22 samples (4.37%), followed by mutation in 6 samples (1.19%), and deep deletion in 5 samples (0.99%; Figure 4A). None of the IDO1 copy-number alterations (CNA) seemed to affect IDO1 expression in HNSCC tumors, as we observed similar IDO1 expression in each type of CNA compared to the diploid samples (Figure 4B).

The 504 HNSCC patients from the cBioPortal database were then clustered into the following two groups: altered group, consisting of patients with IDO1 genetic alterations and unaltered group, consisting of patients without IDO1 genetic alteration. The two groups were then compared in terms of OS and DFS. Alterations in IDO1 appeared to have no significant effect on either OS (Figure 4C) or DFS (Figure 4D). However, there appeared to be a trend (*p* = ns) for better patient OS and DFS in the altered patient group, which was difficult to verify statistically due to the low number of cases with altered IDO1.

**Figure 5:**
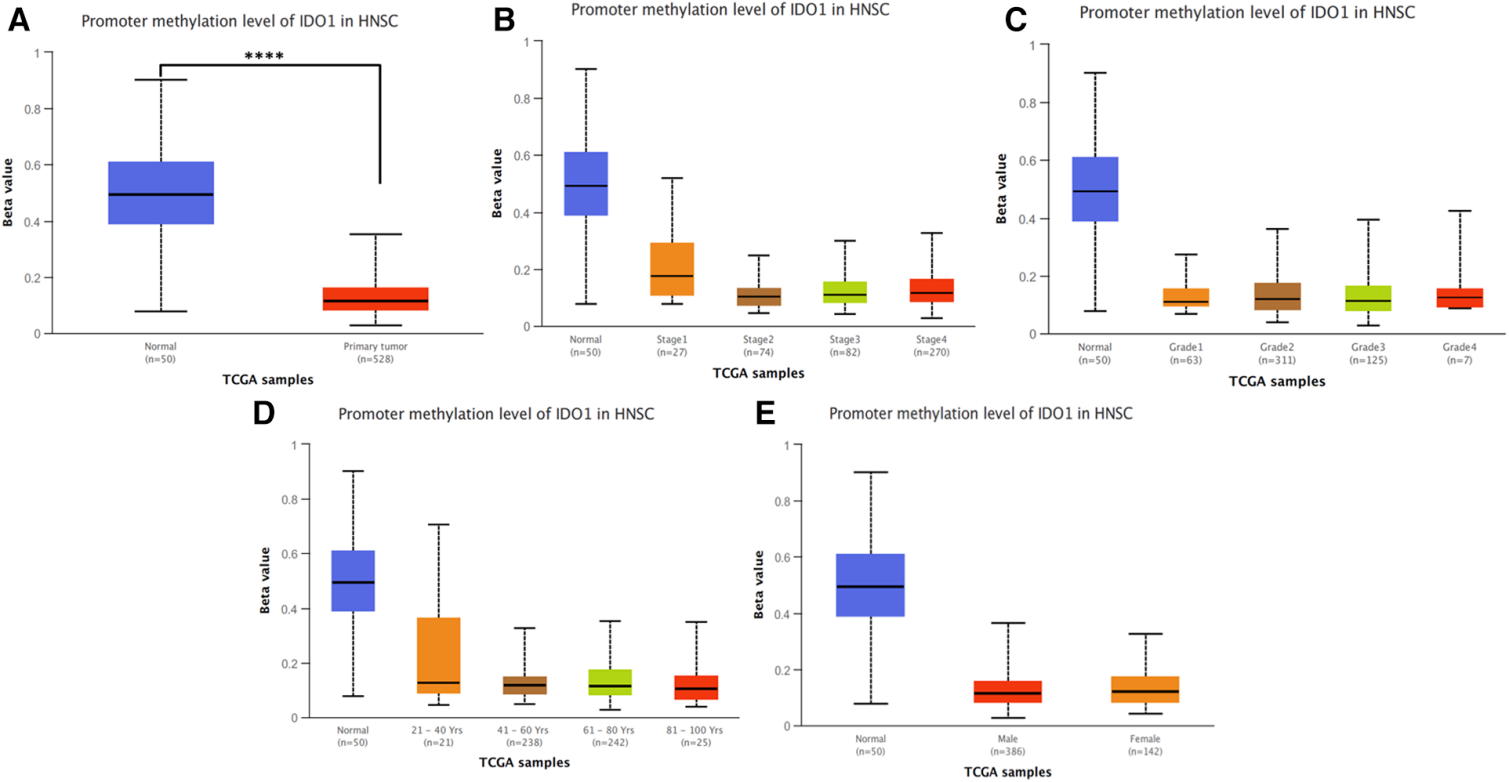
IDO1 DNA promoter methylation analysis. (**A**) Promoter methylation levels of normal tissues and HNSCC tumor samples. Correlation between promoter methylation levels and (**B**) tumor stage, (**C**) tumor grade, (**D**) patient age, and (**E**) patient gender. Statistical significance is provided by UALCAN and is represented by the number of stars; **** *p*-value < 0.0001. (**B–E**) Normal samples were not included in the statistical analysis. HNSCC, head and neck squamous cell carcinoma.

Analysis of IDO1 DNA methylation in UALCAN showed a significant (*p* < 0.0001) decrease in promoter methylation in HNSCC tumor samples (*n* = 528) compared to the adjacent normal tissues (*n* = 50; Figure 5A). Correlation analysis between promoter methylation and tumor stage and grade and patient age and gender did not reveal significant results (Figures 5B–E).

### 3.3 IDO1 expression correlate with the potency of several drugs on HNSCC cell lines

Using the “GDSC gene expression—drug sensitivity correlations” tool of Tableau Public, 15 drugs tested in 40 HNSCC cell lines were significantly correlated with IDO1 expression (Table 2). Drug sensitivity of 2 out of 15 drugs (MG-132 and Z-LLNIe-CHO) was positively correlated with IDO1 expression, while sensitivity of 13 out of 15 drugs (AZD6482, UNC1215, BX-912, OSI-930, CAL-101, XMD13-2, PLX4720, GW-2580, DMOG, BX-795, Genentech Cpd 10, GSK-650394, and QS11) was negatively correlated with IDO1 expression (Table 2).

**Table 2:** Correlation analysis between IDO1 expression levels and drug sensitivity on HNSCC cell lines. Green lines represent positive and red lines negative correlated drugs.

GDSC Drug	Target(s)	IDO1 Correlation (Spearman Rho)	GDSC *p*-value	Clinical Trial for Cancers	Clinical Trial for HNSCC
MG-132	Proteasome	0.9	0.03739	No	No
Z-LLNIe-CHO	γ-secretase	0.9	0.03739	No	No
AZD6482 (KIN-193)	PI3K*β* (P3C2B)	−0.297	0.00546	No	No
UNC1215	L3MBTL3	−0.416	0.0069	No	No
BX-912	PDK1 (PDPK1)	−0.388	0.01122	No	No
OSI-930	KIT, VEGFR, PDGFR	−0.363	0.01816	Yes	No
CAL-101 (idelalisib)	PI3K*δ*	−0.358	0.01977	Yes	No
XMD13-2	RIPK	−0.355	0.02093	No	No
PLX4720	BRAF	−0.25	0.02627	No	No
GW-2580	cFMS	−0.341	0.02731	No	No
DMOG	Prolyl-4-Hydroxylase	−0.326	0.03073	No	No
BX-795	TBK1, PDPK1, IKK, AURKB, AURKC	−0.348	0.03492	No	No
Genentech Cpd 10	AURKA, AURKB	−0.322	0.0373	No	No
GSK-650394	SGK3	−0.313	0.03845	No	No
QS11	ARFGAP	−0.311	0.03957	No	No

GDSC, genomics of drug sensitivity in cancer; HNSCC, head and neck squamous cell carcinoma.

Furthermore, the “clinical trials” tool provided by the U.S. National Library of Medicine (https://clinicaltrials.gov/) was used to explore whether these drugs have been or are currently in clinical trials for different cancers, including HNSCC. Two of these drugs, OSI-930 and idelalisib (CAL-101), have been in clinical trials for other types of cancers. None of the drugs has been in a clinical trial for HNSCC (Table 2).

### 3.4 IDO1 expression is positively correlated with immune-related genes and immune checkpoints in HNSCC

**Figure 6:**
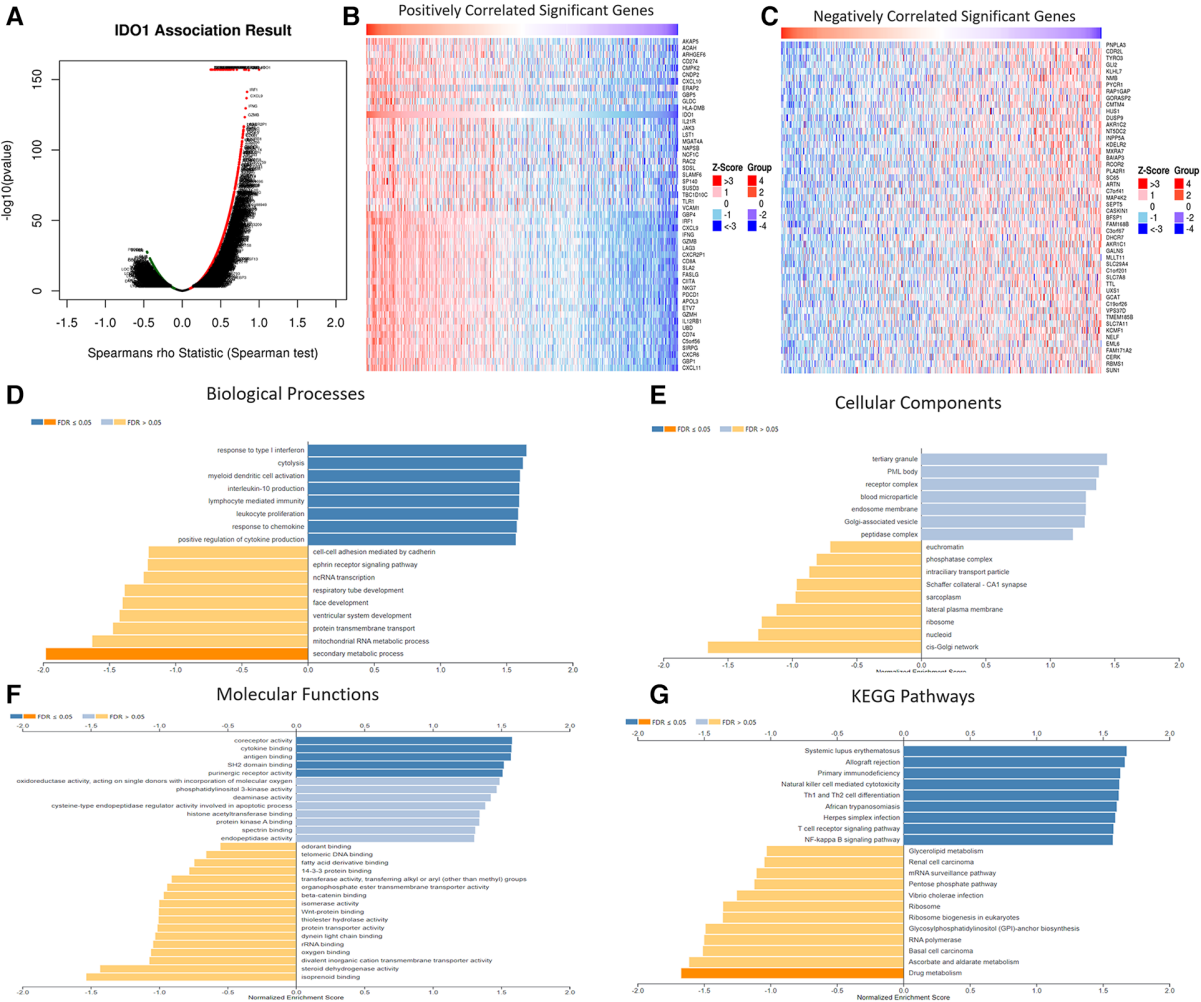
Correlation between IDO1 expression and its co-expressed genes in HNSCC. (**A**) Volcano plot of IDO1-correlated genes in HNSCC. (**B**) Top 50 IDO1 positively correlated genes in HNSCC. (**C**) Top 50 IDO1 negatively correlated genes in HNSCC. (**D**) GO analysis for biological processes of IDO1-correlated genes in HNSCC. (**E**) GO analysis for cellular components of IDO1-correlated genes in HNSCC. (**F**) GO analysis for molecular functions of IDO1-correlated genes in HNSCC. (**G**) GO analysis for the KEGG pathways of IDO1-correlated genes in HNSCC. GO, gene ontology; FDR, false discovery rate; KEGG, kyoto encyclopedia of genes and genomes.

Correlation between IDO1 expression and 20,164 other genes in HNSCC was analyzed in LinkedOmics and revealed that IDO1 is significantly correlated with 10,322 genes expressed in HNSCC (Figure 6A). Of these, 4,715 genes were positively correlated with IDO1 expression, while 5,607 genes were negatively correlated with IDO1 expression. For further analysis, we plotted the top 50 genes positively or negatively correlated with IDO1 expression in HNSCC (Figures 6B,C). Of note, 41 out of the 50 genes that were positively correlated with IDO1 expression (Figure 6B) were immune-related genes, while only 3 out of 50 genes that were negatively correlated with IDO1 expression (Figure 6C) had immune-related roles. Furthermore, GO analysis of these genes revealed that IDO1-correlated genes are mainly involved in immune-related processes, as seen in the analysis of biological processes (Figure 6D), cellular components (Figure 6E), molecular functions (Figure 6F), and KEGG pathways (Figure 6G).

**Figure 7:**
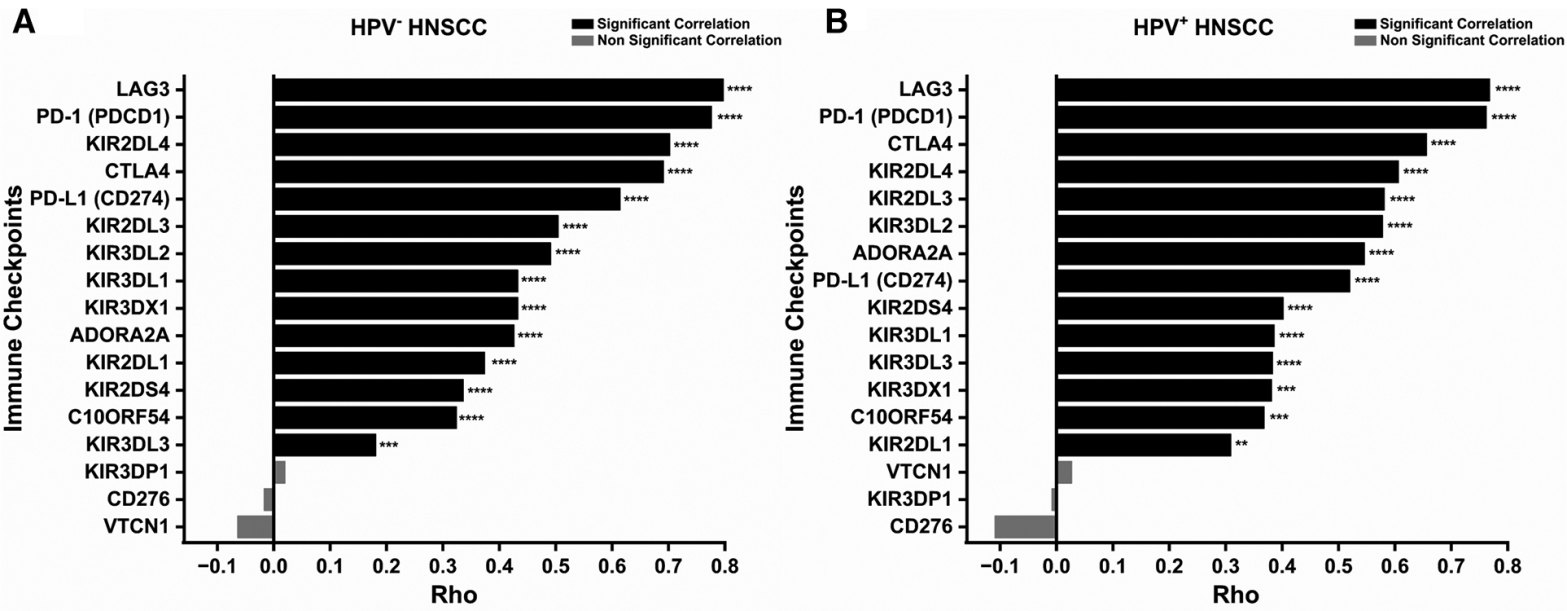
Correlation between IDO1 expression and immune checkpoints in HNSCC. (**A**) Correlation between IDO1 expression and immune checkpoints in HPV− HNSCC. (**B**) Correlation between IDO1 expression and immune checkpoints in HPV+ HNSCC. **p-value < 0.01; ***p-value < 0.001; ****p-value < 0.0001. HNSCC, head and neck squamous cell carcinoma; HPV, Human papillomavirus.

Expression of several immune checkpoints in correlation to IDO1 expression in HNSCC was analyzed with TIMER2.0. IDO1 expression was positively and significantly (at least *p* < 0.01) correlated with most (14 out of 17) of the immune checkpoints tested (Figure 7). Interestingly, when HNSCC cases were stratified by HPV− and HPV+ status, both groups showed similar results (Figures 7A,B).

### 3.5 IDO1 expression positively correlates with immune-cell infiltration in HNSCC

**Figure 8:**
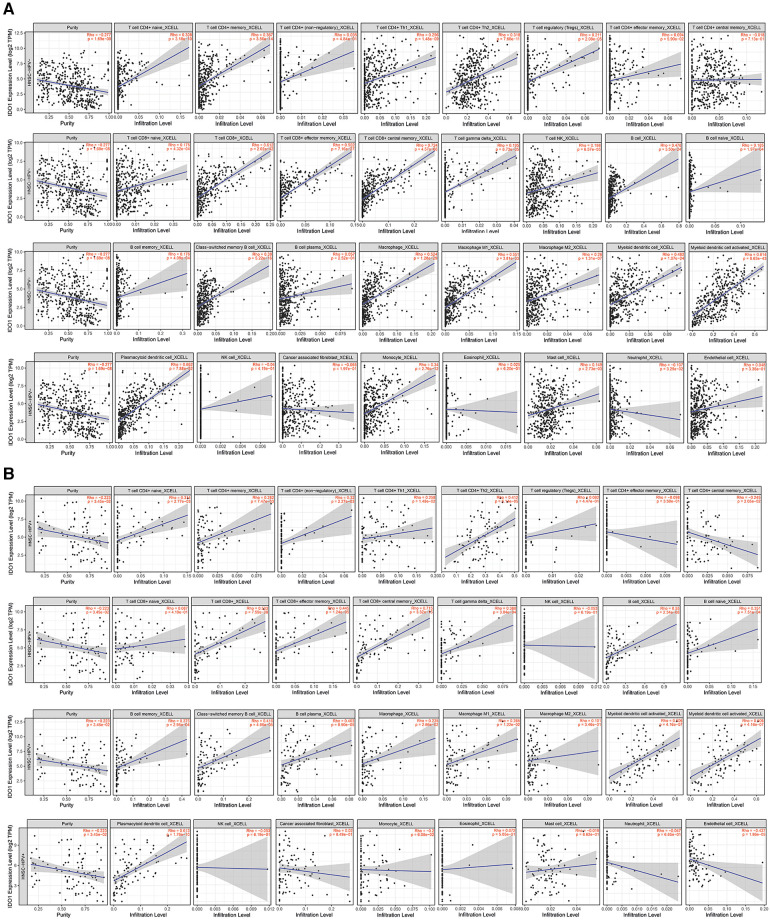
Correlation between IDO1 expression levels and immune infiltration. (**A**) HPV− HNSCC, (**B**) HPV+ HNSCC. Tumor purity for each category (HPV- and HPV+) is depicted in the first place of each line.

**Figure 9:**
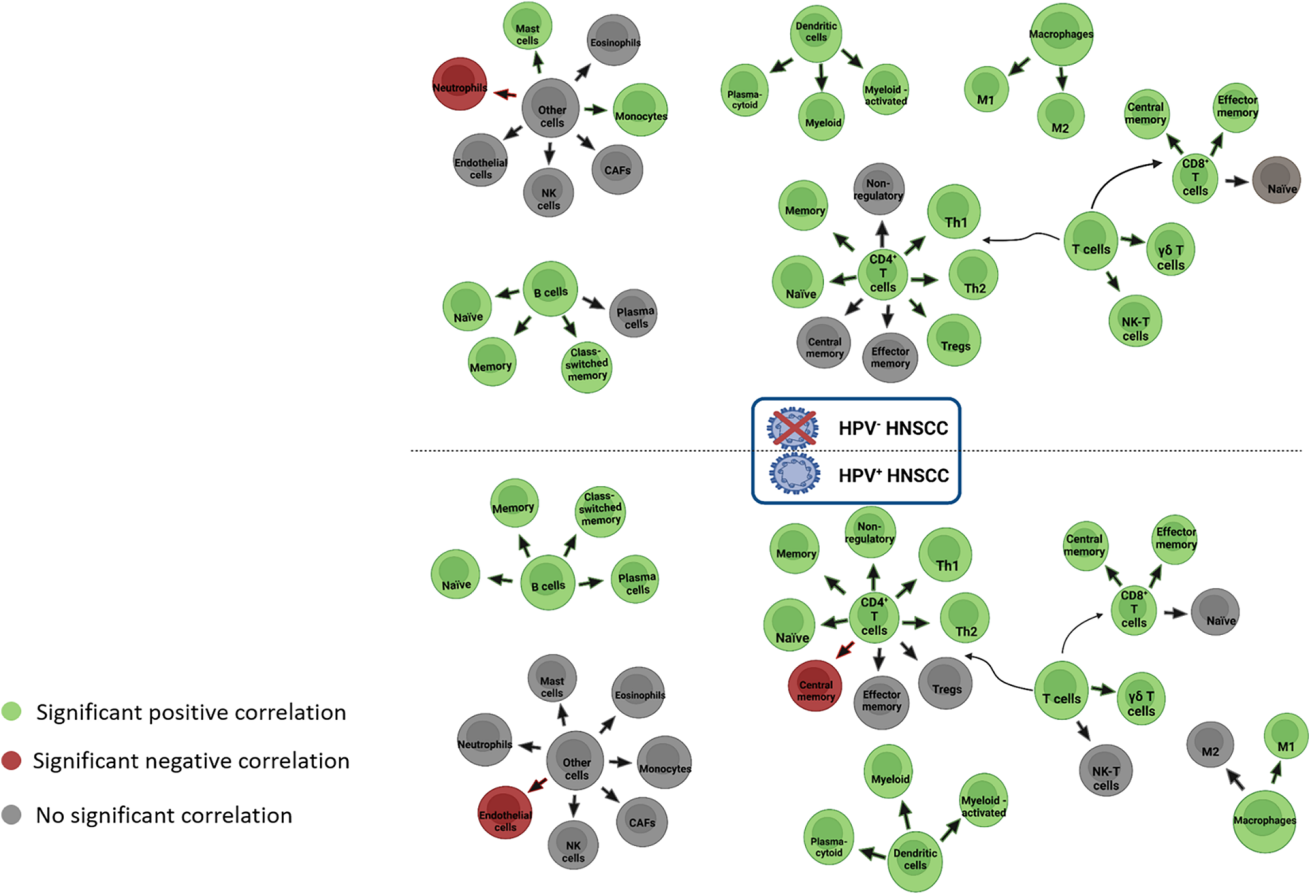
Correlation between IDO1 expression and immune infiltration in HPV− and HPV+ HNSCC. CAFs, cancer-associated fibroblasts; HNSCC, head and neck squamous cell carcinoma; HPV, human papillomavirus; M1/M2, macrophage 1 or macrophage 2; NK, natural killer cells; Th, T helper cells.

Correlation analysis between immune-cell infiltrates and IDO1 expression using TIMER2.0 showed that IDO1 has a significant positive correlation with most of the immune cells in HPV− and HPV+ HNSCC (Figures 8, 9). Interestingly, both groups shared large similarities.

More specifically, in HPV− tumors (Figure 8A), IDO1 seems to be positively correlated with naïve, memory, and class-switched memory B cells; naïve, memory, helper 1, helper 2, and regulatory CD4+ T cells; central memory and effector memory CD8+ T cells; γδ T cells and NK-T cells; plasmacytoid, myeloid, and activated myeloid dendritic cells (DCs); M1 and M2 macrophages; monocytes; and mast cells. IDO1 seems to be negatively correlated with neutrophils in HPV− tumors. In HPV + tumors (Figure 8B), IDO1 appears to be positively correlated with naïve, memory, class-switched memory B cells and plasma cells; naïve, memory, helper 1, helper 2, and non-regulatory CD4+ T cells; central memory and effector memory CD8+ T cells; γδ T cells; plasmacytoid, myeloid, and activated myeloid DCs; and M1 macrophages. In the same cases, IDO1 appears to be negatively correlated with central memory CD4+ T cells and endothelial cells.

## 4 Discussion

IDO1 is highly expressed in a variety of cancers (24). Our analysis revealed that IDO1 expression is higher in HNSCC tumors compared to that in normal tissues. Interestingly, IDO1 is highly expressed especially in HPV+ HNSCC tumors compared with HPV− tumors. The high expression of IDO1 in HPV+ tumors can be explained in the context of high inflammation usually seen in this type of tumor in comparison with HPV− cases (25). We also conducted a comparison analysis of IDO1 expression levels in different cells between the primary tumor and metastatic lymph nodes. This analysis revealed that cancer cells in metastatic lymph nodes express higher levels of IDO1 than cancer cells in the primary tumor. However, based on our IHC staining, this was not validated which could be due to the small sample size and the small difference that was found at the mRNA level. Furthermore, we also observed significantly higher expression of IDO1 in primary tumor mast cells (*N* = 113) than in mast cells located in the metastatic lymph nodes (*N* = 7). However, this difference needs further validation due to the large difference in the number of cells between the two groups.

High IDO1 expression positively correlates with advanced stage in oral squamous cell carcinoma (26). In this study, we investigated the correlation between IDO1 expression and clinicopathological characteristics of HNSCC patients. Our results contrast with those of Struckmeier et al. (26), as higher IDO1 expression is positively associated with lower HNSCC stage. This suggests that IDO1 can be used as a biomarker for new treatment approaches for late-stage HNSCC.

The correlation between IDO1 expression and patient survival is controversial. For example, higher IDO1 expression correlated with better survival in melanoma patients, whereas high IDO1 expression predicted poorer survival in patients with glioblastoma when compared with patients with low IDO1 expression (24). Based on our data, IDO1 expression showed weak to no potential to predict patient survival in HNSCC. Our observation is consistent with previous studies. One study reported that high IDO1 expression has prognostic potential (14), while another study indicated that IDO1 could be used as a prognostic marker only in the early stages of oral cancer (27). In that study, patients with higher IDO1 expression levels had significantly better OS than patients with lower IDO1 levels. However, here the correlation between IDO1 expression and OS was observed only in a univariate analysis, with borderline significance (*p* = 0.047). Additionally, IDO1 expression had no correlation with DFS. Altogether, current data suggest that IDO1 is not a suitable prognostic marker for HNSCC patient survival or for cancer recurrence. Furthermore, we investigated IDO1 genetic alterations and their association with HNSCC patient survival. We found that IDO1 is rarely altered in HNSCC and none of the alterations had any significant effect on IDO1 expression. Moreover, patient survival was similar between patients with (altered group) or without (unaltered group) IDO1 genetic alterations, suggesting that IDO1 genetic changes should not be used as a prognostic marker.

We explored IDO1 DNA promoter methylation in HNSCC and its correlation with patient clinicopathological characteristics. Although no significant correlation was observed between IDO1 DNA promoter methylation and patient characteristics, we found that HNSCC samples have significantly lower IDO1 promoter methylation levels than normal tissues. IDO1 promoter methylation is negatively correlated with IDO1 expression in HNSCC, and HNSCC tumors, especially HPV+, have lower IDO1 promoter methylation (28), consistent with the data presented in this study. In addition, Sailer et al. (27) also showed that IDO1 promoter flank methylation correlates with tumor immunity of HNSCC tissues, as promoter flank methylation is associated with TILs and other immune cells (28). This suggests that IDO1 promoter methylation could be used as a biomarker for immune hot or cold tumors and therefore could be applied for decisions on patient treatment.

An assessment of drug sensitivity in relation to IDO1 expression in 40 HNSCC cell lines revealed two compounds, MG-132 and Z-LLNIe-CHO, that were positively correlated to IDO1 expression. Our analysis showed that 13 compounds had a negative association in terms of drug sensitivity to IDO1 expression. Of these, only two [OSI-930 and CAL-101 (idelalisib)] have been in clinical trials for cancers, but none for HNSCC. Idelalisib has been studied mainly in hematological and B-cell malignancies, while OSI-930 has been studied for advanced solid tumors (NCT02739360, NCT01796470, NCT00513851). These results may be a useful tool for future clinical trials or treatment decisions for patients with high or low IDO1 expression and could open possibilities for more personalized treatment combinations depending on IDO1 expression levels.

IDO1 is significantly positively correlated to genes with immune-related functions, further strengthening the role and relation of IDO1 to tumor immunity. Additionally, we found that IDO1 expression is positively correlated with 14 out of 17 tested immune checkpoints. It is worth mentioning that three of these immune markers belong to the main immune checkpoint pathways in HNSCC, namely PD-1, CTLA-4, and LAG3, which suggests that combination treatment with IDO1 inhibitors may be beneficial for some patients.

We observed that IDO1 expression is positively correlated to most of the immune infiltrates in HNSCC in both HPV+ and HPV− tumors. This correlation is mainly returned to the fact that IDO1 is positively correlated with the presence of cancer-associated inflammation. Additionally, due to M1 and M2 plasticity, it is expected to see a positive correlation between IDO1 and both M1 and M2 at the same time. Thus far, there are not many studies on the association between IDO1 expression and immune infiltration in HNSCC. One of the existing, webtool-based studies conducted by Li et al. showed that IDO1 expression was positively correlated with infiltration of CD4+ T cells, CD8+ T cells, neutrophils, macrophages, and myeloid dendritic cells (14). While our results are slightly different from the study, we stratified HNSCC tumors into HPV− and HPV+ status, which may result in slightly different but more accurate results. Moreover, online databases are constantly updated with new patient samples and data, which can also lead to inconsistent results between different studies.

There are some limitations to the present study. Firstly, most of the acquired data and analyses were based on online databases and web tools. The patient sample number in some of the analyses was small, especially when subcategorizing patients into smaller groups, for example in the analysis of genetic alterations of IDO1 in comparison to HNSCC patient survival. Furthermore, the treatment approach of HNSCC patients was not reported and was not considered for any of the analyses.

In conclusion, IDO1 appears to be highly associated with the immune landscape in HNSCC patients and is therefore a promising target for new treatment approaches. Further elucidating the association between IDO1, TME, and immune profile of HNSCC may be essential for identifying predictive biomarkers that could be useful in personalized immunotherapeutic approaches. Further *in vitro* and *in vivo* experiments are needed for a more in-depth knowledge on the biology of IDO1 in HNSCC.

## Data Availability

The datasets presented in this study can be found in online repositories. The names of the repository/repositories and accession number(s) can be found in the article/Supplementary Material.
